# 3,5-Dichloro-6-methyl­pyridin-2-amine

**DOI:** 10.1107/S1600536808041366

**Published:** 2008-12-13

**Authors:** Hoong-Kun Fun, Reza Kia, Annada C. Maity, Sibaprasad Maity, Shyamaprosad Goswami

**Affiliations:** a X-ray Crystallography Unit, School of Physics, Universiti Sains Malaysia, 11800 USM, Penang, Malaysia; b Department of Chemistry, Bengal Engineering and Science University, Shibpur, Howrah 711 103, India

## Abstract

In the title compound, C_6_H_6_Cl_2_N_2_, intra­molecular N–H⋯Cl and C—H⋯Cl contacts generate five-membered rings, producing *S*(5) ring motifs. Pairs of inter­molecular N—H⋯N hydrogen bonds link neighbouring mol­ecules into dimers with *R* ^2^_2_(8) ring motifs. In the crystal structure, these dimers are connected by N—H⋯Cl inter­actions and are packed into columns.

## Related literature

For details of hydrogen-bond motifs, see: Bernstein *et al.* (1995 ▶). For related literature and applications see, for example: Goswami & Maity (2007 ▶); Taylor *et al.* (1989 ▶); Taylor & Ray (1988 ▶); Beer *et al.* (1993 ▶); Goswami *et al.* (2000 ▶, 2005 ▶); Fun *et al.* (2008 ▶).
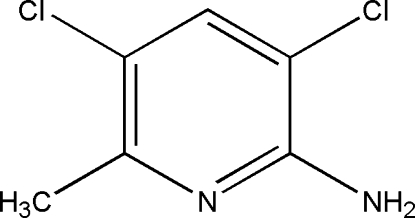


## Experimental

### 

#### Crystal data

C_6_H_6_Cl_2_N_2_
*M* *_r_* = 177.03
Monoclinic, 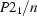

*a* = 12.7670 (3) Å
*b* = 3.8037 (1) Å
*c* = 15.4129 (3) Å
β = 104.990 (1)°
*V* = 723.01 (3) Å^3^
*Z* = 4
Mo *K*α radiation
μ = 0.81 mm^−1^
*T* = 100.0 (1) K
0.45 × 0.31 × 0.28 mm

#### Data collection

Bruker SMART APEXII CCD area-detector diffractometer
Absorption correction: multi-scan (**SADABS**; Bruker, 2005 ▶) *T*_min_ = 0.711, *T*_max_ = 0.803
26531 measured reflections
3795 independent reflections
3485 reflections with *I* > 2˘*I*)
*R*_int_ = 0.026

#### Refinement

*R*[*F* ^2^ > 2σ(*F* ^2^)] = 0.024
*wR*(*F* ^2^) = 0.071
*S* = 1.11
3795 reflections
115 parameters
All H-atom parameters refined
Δρ_max_ = 0.61 e Å^−3^
Δρ_min_ = −0.42 e Å^−3^

### 

Data collection: *APEX2* (Bruker, 2005 ▶); cell refinement: *SAINT* (Bruker, 2005 ▶); data reduction: *SAINT*; program(s) used to solve structure: *SHELXTL* (Sheldrick, 2008 ▶); program(s) used to refine structure: *SHELXTL*; molecular graphics: *SHELXTL*; software used to prepare material for publication: *SHELXTL* and *PLATON* (Spek, 2003 ▶).

## Supplementary Material

Crystal structure: contains datablocks global, I. DOI: 10.1107/S1600536808041366/tk2343sup1.cif

Structure factors: contains datablocks I. DOI: 10.1107/S1600536808041366/tk2343Isup2.hkl

Additional supplementary materials:  crystallographic information; 3D view; checkCIF report

## Figures and Tables

**Table 1:** Hydrogen-bond geometry (Å, °)

*D*—H⋯*A*	*D*—H	H⋯*A*	*D*⋯*A*	*D*—H⋯*A*
N2—H2*N*1⋯N1^i^	0.869 (15)	2.168 (15)	3.0320 (9)	172.8 (13)
N2—H1*N*1⋯Cl2	0.828 (14)	2.603 (14)	3.0156 (7)	112.3 (12)
C6—H6*A*⋯Cl1	1.02 (2)	2.67 (2)	3.1318 (9)	108.0 (14)
N2—H1*N*1⋯Cl2^ii^	0.828 (14)	2.900 (14)	3.6758 (7)	156.9 (13)

Symmetry code: (i) 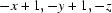.

**Fig. 1.**
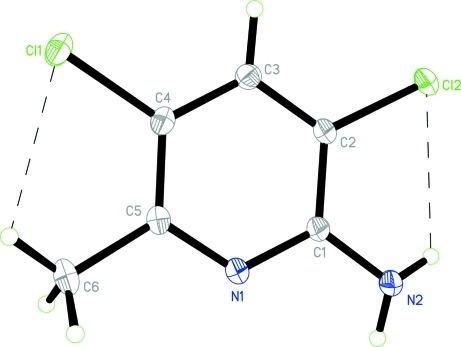
The molecular structure of (I), showing 50% probability displacement ellipsoids and the atomic numbering. Dashed lines show intramolecular hydrogen bonds.

**Fig. 2.**
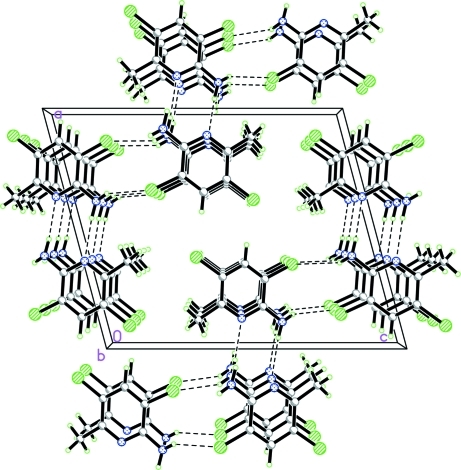
The crystal packing for (I), showing dimers with R22(8) motifs and stacking of the dimers into columns along the b-axis. Intermolecular interactions are drawn as dashed lines.

## supplementary crystallographic information

### Comment

The halogen substituted π-depleted heteroaromatics (*e.g.* pterin, quinoxaline, naphthyridine, or pyridine derivatives) are important intermediates in modern organic chemistry (Goswami & Maity 2007; Taylor *et al.* 1989; Taylor & Ray 1988; Beer *et al.* 1993), e.g. they are used as precursors for pharmacologically active compounds. These are also versatile compounds in manifold synthesis of artificial receptors for molecular recognition (Goswami *et al.* 2000, 2005; Fun *et al.* 2008).

In the title compound (I), Fig. 1, intramolecular N–H···Cl and C—H···Cl contacts generate five-membered rings, producing *S(5)* ring motifs (Bernstein *et al.*, 1995). Pairs of intermolecular N—H···N hydrogen bonds link molecules into dimers with a *R^2^_2_(8)* ring motif (Table 1). In the crystal structure, these dimers are connected by N—H···Cl interactions and are packed into columns along the *b* axis, Fig. 2.

### Experimental

Phosphorus oxychloride (POCl_3_) (15 ml) was added to 2-amino-6-methylpyridine (2 g, 0.019 mmol) and the mixture was refluxed at 383 K for 16 h. Excess POCl_3_ was distilled off. The solid residue was neutralized using KOH solution in an ice bath and a saturated NaHCO_3_ solution was added. The solid residue was filtered off, extracted with CHCl_3_, the solution was dried over anhydrous Na_2_SO_4_ and then concentrated under vacuum. The crude product was purified by column chromatography using silica gel with 20% ethyl acetate in petroleum ether as eluant to afford (I) (2.14 g, 65%) as a colourless crystalline solid, mp. 404–407 K.

### Refinement

All hydrogen atoms were located from a difference Fourier map and refined freely; range of C-H distances: 0.924 (7) to 1.02 (2) Å. See Table 1 for N-H distances.

### Crystal data

**Table:**

C_6_H_6_Cl_2_N_2_	*F*(000) = 360
*M**_r_* = 177.03	*D*_x_ = 1.626 Mg m^−^^3^
Monoclinic, *P*2_1_/*n*	Melting point: 404 K
Hall symbol: -P 2yn	Mo *K*α radiation, λ = 0.71073 Å
*a* = 12.7670 (3) Å	Cell parameters from 9896 reflections
*b* = 3.8037 (1) Å	θ = 2.4–40.3°
*c* = 15.4129 (3) Å	µ = 0.81 mm^−^^1^
β = 104.990 (1)°	*T* = 100 K
*V* = 723.01 (3) Å^3^	Block, colourless
*Z* = 4	0.45 × 0.31 × 0.28 mm

### Data collection

**Table:**

Bruker SMART APEXII CCD area-detector diffractometer	3795 independent reflections
Radiation source: fine-focus sealed tube	3485 reflections with *I* > 2˘*I*)
graphite	*R*_int_ = 0.026
φ and ω scans	θ_max_ = 37.5°, θ_min_ = 2.4°
Absorption correction: multi-scan (*SADABS*; Bruker, 2005)	*h* = −21→21
*T*_min_ = 0.711, *T*_max_ = 0.803	*k* = −6→6
26531 measured reflections	*l* = −26→26

### Refinement

**Table:**

Refinement on *F*^2^	Primary atom site location: structure-invariant direct methods
Least-squares matrix: full	Secondary atom site location: difference Fourier map
*R*[*F*^2^ > 2σ(*F*^2^)] = 0.024	Hydrogen site location: inferred from neighbouring sites
*wR*(*F*^2^) = 0.071	All H-atom parameters refined
*S* = 1.11	*w* = 1/[σ^2^(*F*_o_^2^) + (0.037*P*)^2^ + 0.1631*P*] where *P* = (*F*_o_^2^ + 2*F*_c_^2^)/3
3795 reflections	(Δ/σ)_max_ = 0.001
115 parameters	Δρ_max_ = 0.61 e Å^−^^3^
0 restraints	Δρ_min_ = −0.42 e Å^−^^3^

### Special details

**Table:**

Experimental. The low-temperature data was collected with the Oxford Cyrosystem Cobra low-temperature attachment.
Geometry. All e.s.d.'s (except the e.s.d. in the dihedral angle between two l.s. planes) are estimated using the full covariance matrix. The cell e.s.d.'s are taken into account individually in the estimation of e.s.d.'s in distances, angles and torsion angles; correlations between e.s.d.'s in cell parameters are only used when they are defined by crystal symmetry. An approximate (isotropic) treatment of cell e.s.d.'s is used for estimating e.s.d.'s involving l.s. planes.
Refinement. Refinement of *F*^2^ against ALL reflections. The weighted *R*-factor *wR* and goodness of fit *S* are based on *F*^2^, conventional *R*-factors *R* are based on *F*, with *F* set to zero for negative *F*^2^. The threshold expression of *F*^2^ > σ(*F*^2^) is used only for calculating *R*-factors(gt) *etc*. and is not relevant to the choice of reflections for refinement. *R*-factors based on *F*^2^ are statistically about twice as large as those based on *F*, and *R*- factors based on ALL data will be even larger.

### Fractional atomic coordinates and isotropic or equivalent isotropic displacement parameters (Å^2^)

**Table:**

	*x*	*y*	*z*	*U*_iso_*/*U*_eq_	
Cl1	0.116608 (15)	0.89761 (5)	0.117299 (12)	0.01973 (5)	
Cl2	0.150078 (13)	0.32687 (5)	−0.194719 (10)	0.01603 (4)	
N1	0.36956 (5)	0.65340 (16)	0.02268 (4)	0.01455 (9)	
N2	0.38950 (5)	0.41291 (19)	−0.10983 (4)	0.01738 (11)	
H2N1	0.4571 (12)	0.379 (4)	−0.0819 (9)	0.027 (3)*	
H1N1	0.3607 (11)	0.284 (4)	−0.1526 (9)	0.025 (3)*	
C1	0.32310 (5)	0.51854 (18)	−0.05855 (4)	0.01322 (10)	
C2	0.20904 (5)	0.49965 (17)	−0.08923 (4)	0.01321 (10)	
C3	0.14544 (5)	0.61633 (18)	−0.03563 (4)	0.01465 (10)	
H3	0.0672 (10)	0.605 (3)	−0.0583 (8)	0.022 (3)*	
C4	0.19660 (5)	0.75331 (18)	0.04856 (4)	0.01450 (10)	
C5	0.30903 (5)	0.77194 (18)	0.07629 (4)	0.01437 (10)	
C6	0.37037 (7)	0.9165 (2)	0.16549 (5)	0.02130 (13)	
H6C	0.4268 (13)	1.055 (5)	0.1579 (11)	0.041 (4)*	
H6B	0.4079 (16)	0.744 (5)	0.2083 (12)	0.061 (5)*	
H6A	0.3222 (16)	1.071 (6)	0.1929 (13)	0.065 (6)*	

### Atomic displacement parameters (Å^2^)

**Table:**

	*U*^11^	*U*^22^	*U*^33^	*U*^12^	*U*^13^	*U*^23^
Cl1	0.02170 (8)	0.02155 (8)	0.01862 (8)	0.00364 (6)	0.01006 (6)	−0.00108 (5)
Cl2	0.01665 (7)	0.01786 (7)	0.01167 (7)	−0.00132 (5)	0.00022 (5)	−0.00081 (5)
N1	0.0143 (2)	0.0174 (2)	0.0113 (2)	−0.00002 (17)	0.00219 (17)	−0.00112 (17)
N2	0.0146 (2)	0.0247 (3)	0.0127 (2)	0.00163 (19)	0.00317 (18)	−0.00292 (19)
C1	0.0136 (2)	0.0146 (2)	0.0110 (2)	0.00045 (18)	0.00251 (18)	0.00056 (18)
C2	0.0140 (2)	0.0139 (2)	0.0108 (2)	−0.00021 (18)	0.00163 (17)	0.00039 (18)
C3	0.0143 (2)	0.0153 (2)	0.0142 (2)	0.00085 (19)	0.00352 (19)	0.0011 (2)
C4	0.0168 (2)	0.0140 (2)	0.0137 (2)	0.0020 (2)	0.00586 (19)	0.00056 (19)
C5	0.0172 (2)	0.0141 (2)	0.0117 (2)	0.00022 (19)	0.00350 (19)	−0.00028 (19)
C6	0.0264 (3)	0.0224 (3)	0.0135 (3)	−0.0011 (3)	0.0023 (2)	−0.0044 (2)

### Geometric parameters (Å, °)

**Table:**

Cl1—C4	1.7396 (7)	C2—C3	1.3733 (9)
Cl2—C2	1.7346 (6)	C3—C4	1.3941 (9)
N1—C1	1.3409 (8)	C3—H3	0.970 (13)
N1—C5	1.3468 (9)	C4—C5	1.3894 (10)
N2—C1	1.3607 (9)	C5—C6	1.4996 (10)
N2—H2N1	0.869 (14)	C6—H6C	0.924 (17)
N2—H1N1	0.828 (14)	C6—H6B	0.96 (2)
C1—C2	1.4118 (9)	C6—H6A	1.02 (2)
			
C1—N1—C5	121.03 (6)	C5—C4—C3	120.23 (6)
C1—N2—H2N1	116.4 (9)	C5—C4—Cl1	121.27 (5)
C1—N2—H1N1	115.0 (9)	C3—C4—Cl1	118.50 (5)
H2N1—N2—H1N1	119.1 (13)	N1—C5—C4	120.35 (6)
N1—C1—N2	117.62 (6)	N1—C5—C6	116.03 (6)
N1—C1—C2	120.07 (6)	C4—C5—C6	123.62 (6)
N2—C1—C2	122.28 (6)	C5—C6—H6C	109.4 (10)
C3—C2—C1	120.08 (6)	C5—C6—H6B	115.3 (11)
C3—C2—Cl2	120.37 (5)	H6C—C6—H6B	102.0 (15)
C1—C2—Cl2	119.55 (5)	C5—C6—H6A	111.3 (11)
C2—C3—C4	118.24 (6)	H6C—C6—H6A	107.2 (15)
C2—C3—H3	118.9 (7)	H6B—C6—H6A	110.8 (14)
C4—C3—H3	122.8 (7)		
			
C5—N1—C1—N2	178.27 (6)	C2—C3—C4—C5	0.46 (10)
C5—N1—C1—C2	0.13 (10)	C2—C3—C4—Cl1	−179.37 (5)
N1—C1—C2—C3	−0.61 (10)	C1—N1—C5—C4	0.64 (10)
N2—C1—C2—C3	−178.66 (7)	C1—N1—C5—C6	179.98 (6)
N1—C1—C2—Cl2	179.63 (5)	C3—C4—C5—N1	−0.94 (10)
N2—C1—C2—Cl2	1.58 (9)	Cl1—C4—C5—N1	178.89 (5)
C1—C2—C3—C4	0.29 (10)	C3—C4—C5—C6	179.77 (7)
Cl2—C2—C3—C4	−179.95 (5)	Cl1—C4—C5—C6	−0.40 (10)

### Hydrogen-bond geometry (Å, °)

**Table:**

*D*—H···*A*	*D*—H	H···*A*	*D*···*A*	*D*—H···*A*
N2—H2N1···N1^i^	0.869 (15)	2.168 (15)	3.0320 (9)	172.8 (13)
N2—H1N1···Cl2	0.828 (14)	2.603 (14)	3.0156 (7)	112.3 (12)
C6—H6A···Cl1	1.02 (2)	2.67 (2)	3.1318 (9)	108.0 (14)
N2—H1N1···Cl2^ii^	0.828 (14)	2.900 (14)	3.6758 (7)	156.9 (13)

Symmetry codes: (i) −*x*+1, −*y*+1, −*z*; (ii) −*x*+1/2, *y*−1/2, −*z*−1/2.
